# Supplementary material for: Role of charges in a dynamic disordered complex between an IDP and a folded domain
Source: Nat Commun. 2025 Apr 4;16:3242. doi: 10.1038/s41467-025-58374-5 (PMC11971343; doi:10.1038/s41467-025-58374-5)
Supplement: Supplementary file 1 — Supplementary Information [file 41467_2025_58374_MOESM1_ESM.pdf]

Supplementary information

Role of charges in a dynamic disordered complex between an IDP and a folded domain

Katrine Bugge[1]*, Andrea Sottini[2], Miloš T. Ivanović[2], Freia Buus[1], Daniel Saar[1], Catarina B. Fernandes[1], Fabienne Kocher[2], Jacob H. Martinsen[1], Benjamin Schuler[2,3]*, Robert B. Best[4]*, Birthe B. Kragelund[1]*

SUPPLEMENTARY FIGURES

Supplementary Fig. 1: Secondary chemical shifts of ProTα in the presence of equimolar and eight molar ratio of GD.
Supplementary Fig. 2: NMR titrations of ^{15}N-GD with ProTα
Supplementary Fig. 3: Transfer efficiency plots at different ionic strengths..
Supplementary Fig. 4: Relaxation rates and hetNOEs of ^{15}N-GD-WT with and without ProTα and Bracken plots of ^{15}N-GD-WT relaxation rates.
Supplementary Fig. 5: Comparison of experimental and simulated R_1, R_2, and hetNOE values for ^{15}N-GD:ProTα.
Supplementary Fig. 6: Influence of GD-WT binding on the chain dynamics of ProTα from nsFCS
Supplementary Fig. 7: Comparison of experimental and simulated R_1, R_2, and hetNOE values for ^{15}N-GD:ProTα for several different force fields.
Supplementary Fig. 8: Distributions of lifetimes of intermolecular contacts from GD:ProTα simulations using the Amber ff03ws force field and the des-amber force field.
Supplementary Fig. 9: ^{15}N-HSQC spectra of ^{15}N- ProTα alone and with addition of three different proteins.
Supplementary Fig. 10: Backbone amide CSPs of ^{15}N-ProTα upon addition of GD-WT or GD charge variants.
Supplementary Fig. 11: Quantification of charge variants affinity for ProTα using smFRET.
Supplementary Fig. 12: Urea unfolding of GD.
Supplementary Fig. 13: Backbone amide CSPs of ^{15}N- ProTα upon addition of 4 M urea.
Supplementary Fig. 14: Thermal denaturation of GD-WT and variants.
Supplementary Fig. 15: CSPs of ^{15}N-ProTα upon addition of GD-WT or GD charge swap variants.
Supplementary Fig. 16: CSPs of ^{15}N-ProTα upon addition of GD-WT or GD charge swap variants.
Supplementary Fig. 17: Normalized CSPs (CSP_{sum}; see methods).
Supplementary Fig. 18: CSP_{sum} plotted against apparent K_D.
Supplementary Fig. 19: Apparent K_Ds plotted against net charge of variants.
Supplementary Fig. 20: Illustration of charge clustering in GD charge density variants
Supplementary Fig. 21: Comparison of relaxation parameters calculated from two independent simulations

SUPPLEMENTARY TABLES

Supplementary Table 1: Extraction of K_D values at higher stoichiometries from the CG simulation.
Supplementary Table 2: Summary of simulations performed.

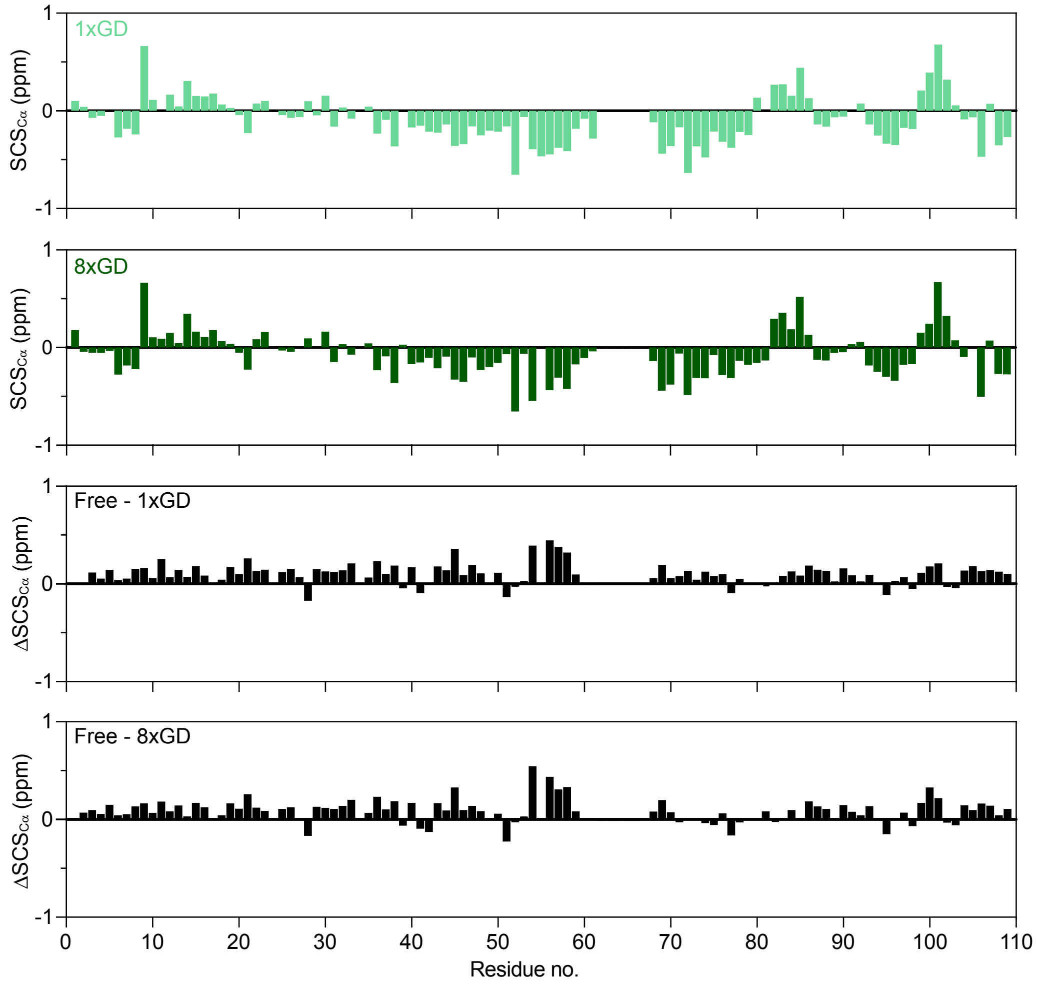

Supplementary Fig. 1: *C^{α} secondary chemical shifts ($SCS_{C\alpha}$) of ProTα in the presence of equimolar concentration of GD (top panel, light green) and at a 8x molar ratio of GD (second panel, dark green). Difference in C^{α} secondary chemical shifts ($\Delta SCS_{C\alpha}$) of ProTα in its free and GD-bound states (equimolar ratio, third panel, and 8x molar ratio, bottom panel)*

Related to Figure 1.

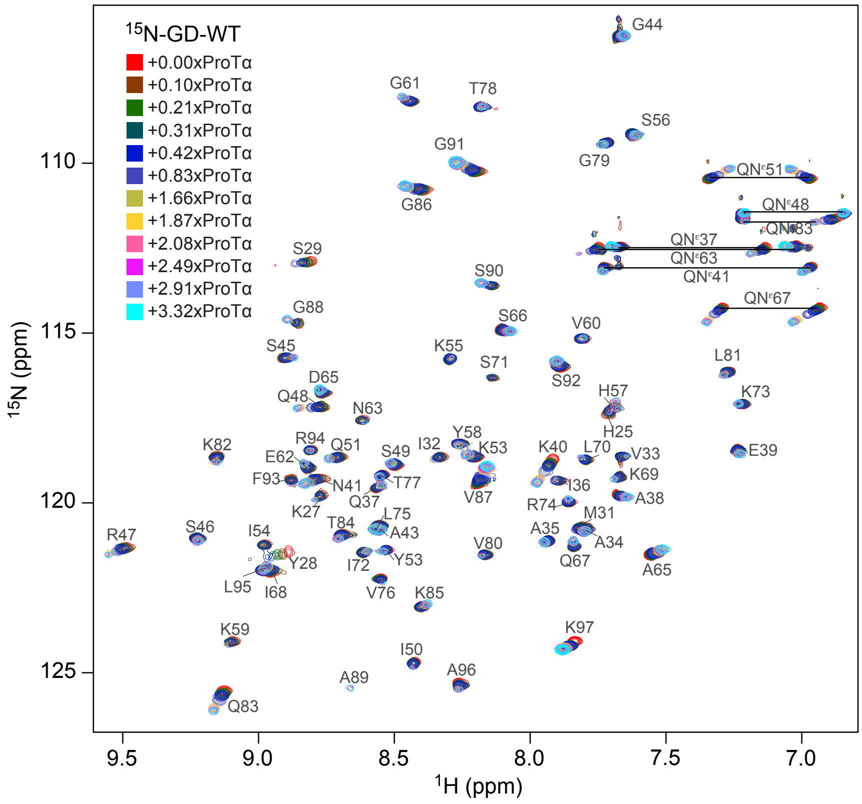

Supplementary Fig. 2: *^{15}N-HSQC spectra of ^{15}N-GD titrated with ProTα at ratios in accordance with the color key. Peak assignments are inserted as labels.*

Related to Figure 2.

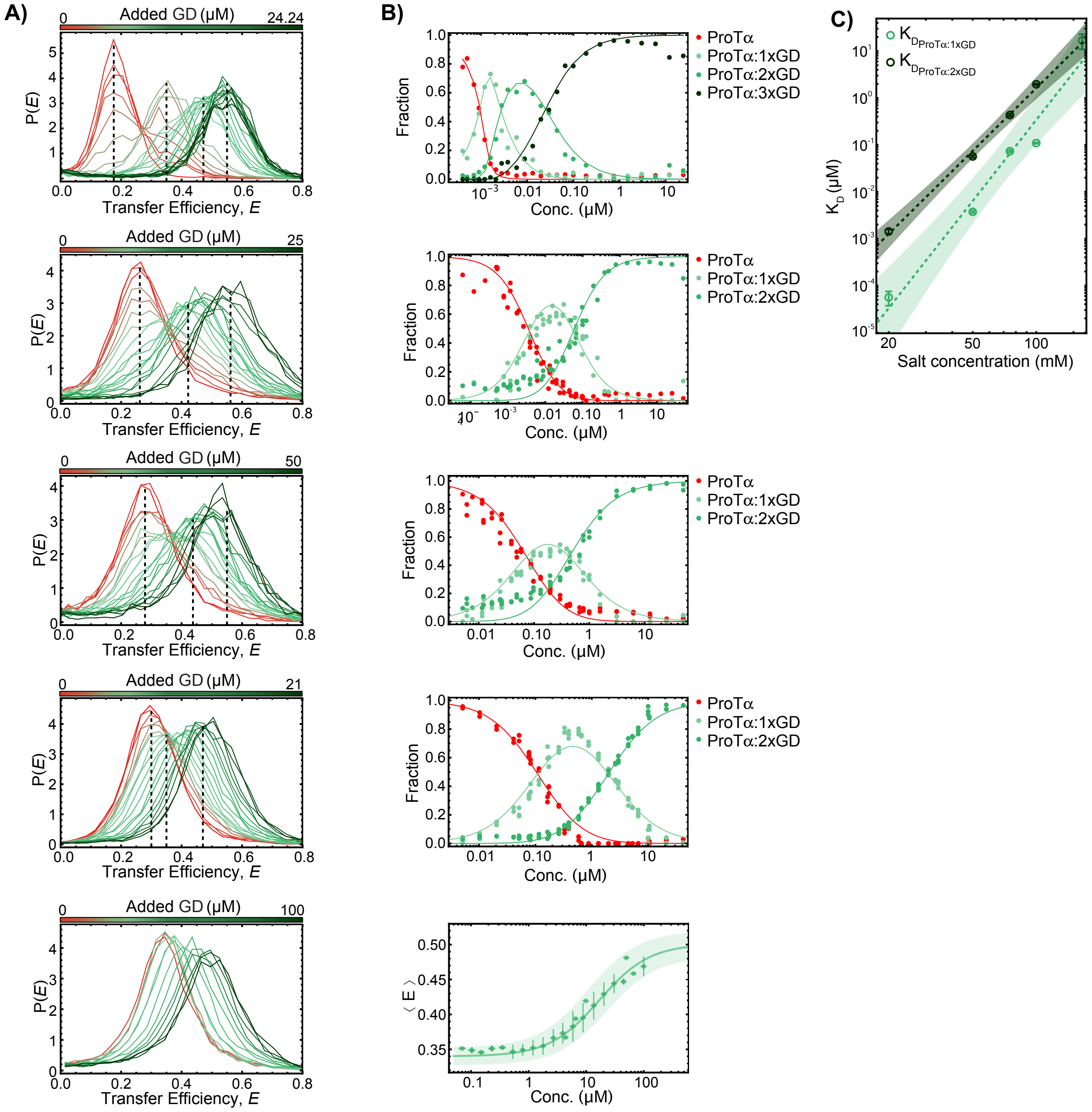

Supplementary Fig. S3: *Interaction of fluorescently labeled ProTα with GD measured at different ionic strengths using smFRET.* ***A)*** *Transfer efficiency histograms from smFRET experiments of labeled ProTα with increasing concentration of GD at five different ionic strengths (IS) (from top to bottom: 20, 50 , 75, 100 and 165 mM). Multiple subpopulations are visible (especially at 20, 50, 75 and 100 mM ionic strength) indicating increasing ProTα compaction upon binding of multiple GD molecules (up to three GD bound to ProTα can be identified at 20mM, whereas only up to two GD molecules bound to ProTα are visible at 50, 75 and 100 mM ionic strength) At an ionic strength of 165 mM, no subpopulations can be identified due to fast exchange between the free and bound states of ProTα. The mean transfer efficiencies of the subpopulations are indicated by vertical dashed lines in each plot.* ***B)*** *Relative fraction of the visible subpopulations in the histograms in panel A) fit with a model describing the equilibrium of ProTα free and bound to up to three GD molecules, at 20 mM ionic strength, or up to two GD molecules, at 50, 75 and 100 mM.* ***C)*** *Affinity of the 1:1 ProTα:GD and 1:2 ProTα:GD complexes as a function of ionic strength. The dashed lines and the shaded areas are the fit and 90% confidence interval of the Record-Lohman fi[1]t describing the counter ion release upon the formation of the complex. Related to Figure 1.*

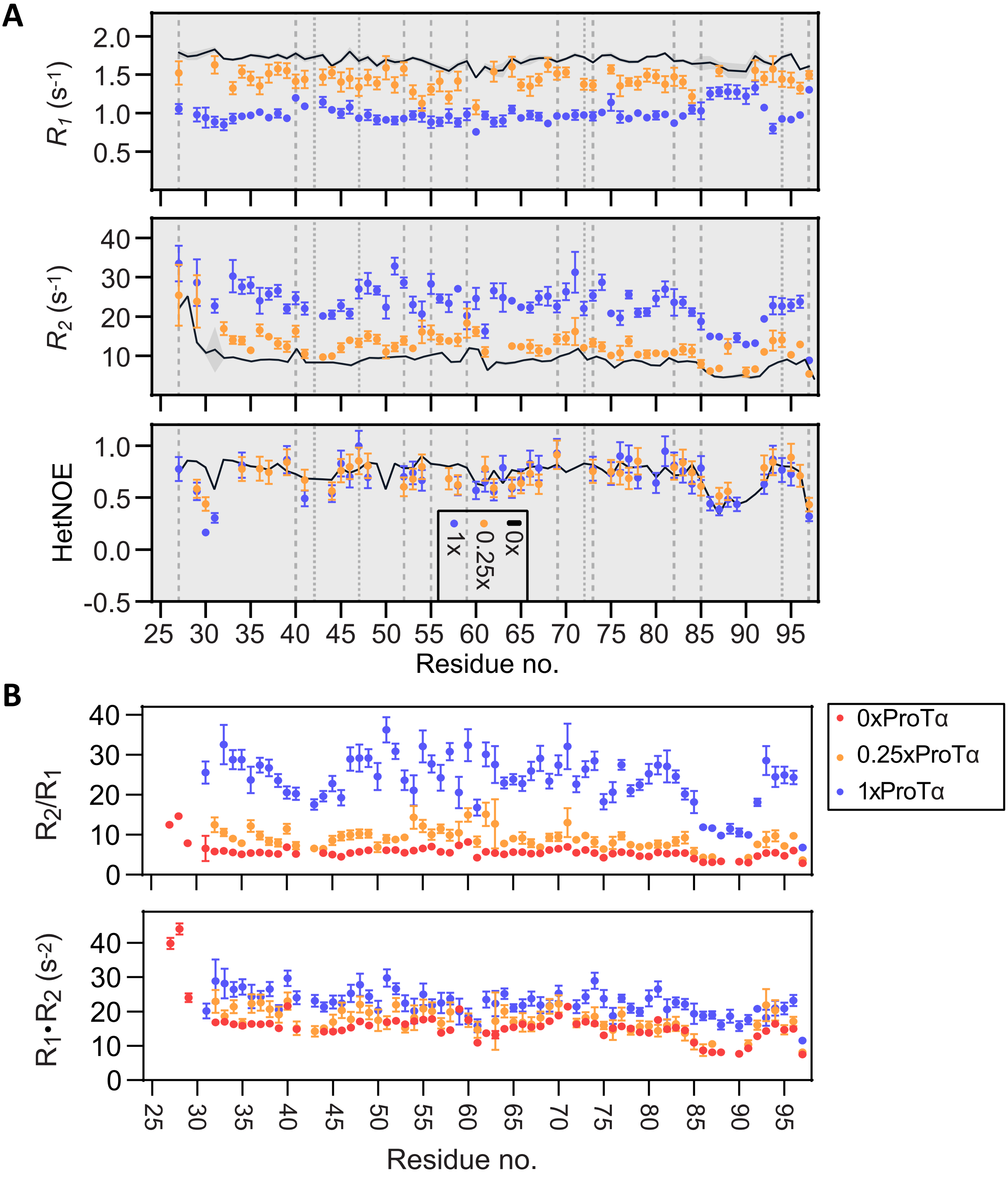

Supplementary Fig. 4*: A)* *R_1 and R_2 relaxation rates and hetNOEs of GD in the absence (black line, data from Martinsen et al.,* [2]*) and the presence of 0.25x and 1x molar ratio of ProTα. The data in the presence of ProTα are the same as shown in Fig. 2D.* ***B)*** *Bracken plots* [3] *of ^{15}N-GD R_1 and R_2 relaxation rates (600 MHz) upon addition of 0-1 times molar ratio of ProTα in accordance with the color key. Erros bars are standard errors of the fits.*

Related to Figure 2.

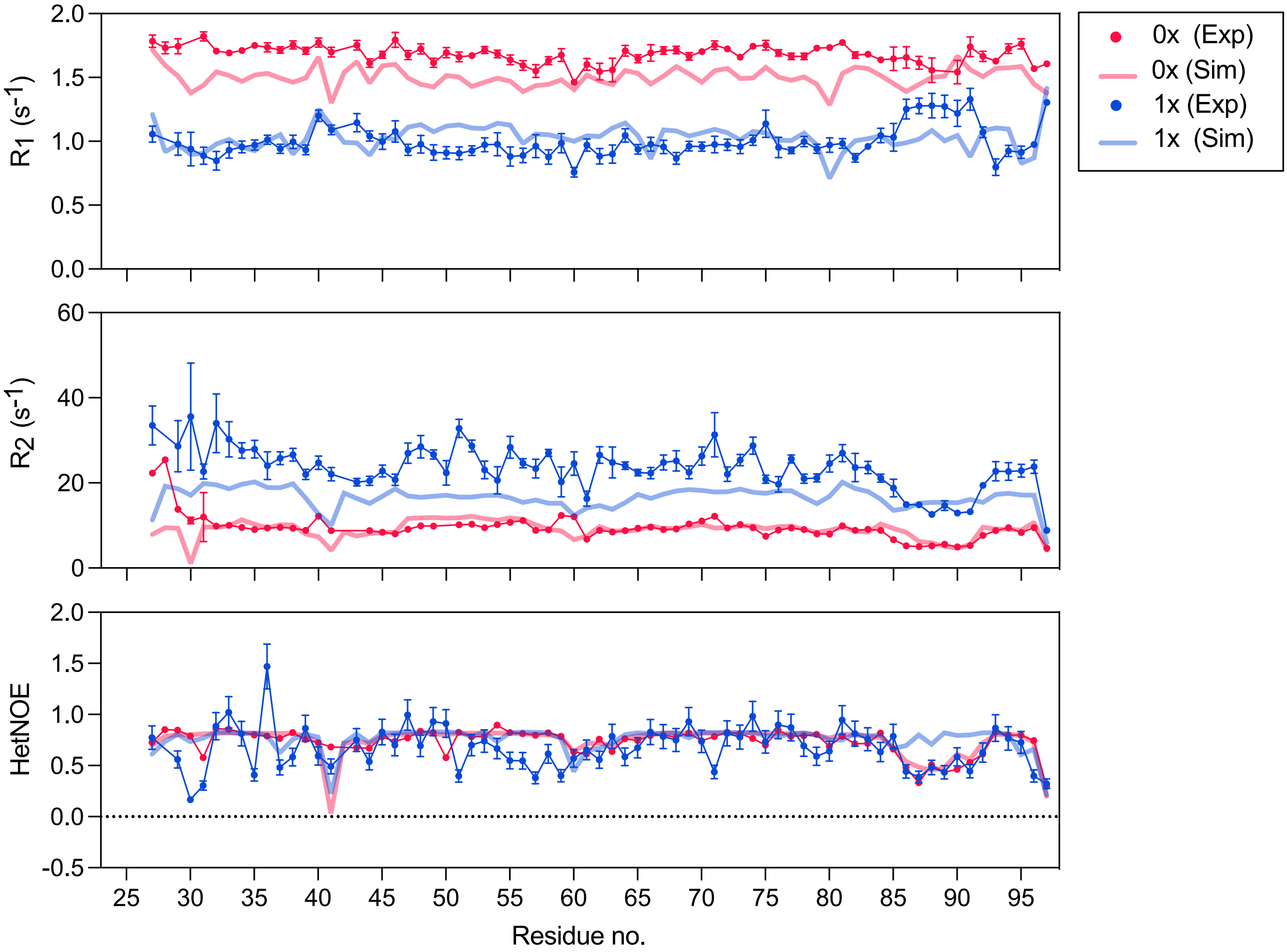

Supplementary Fig. 5*: Comparison of experimentally determined relaxation rates R_1 and R_2, and hetNOEs for free ^{15}N-GD (blue symbols) and equimolar ration of ^{15}N-GD:ProTα (red symbols) and the relaxation rates determined by molecular dynamics simulation with the DES-Amber force field (lines colored correspondingly). Erros barson the experimental data are standard errors of the fits.*

Related to Figure 3.

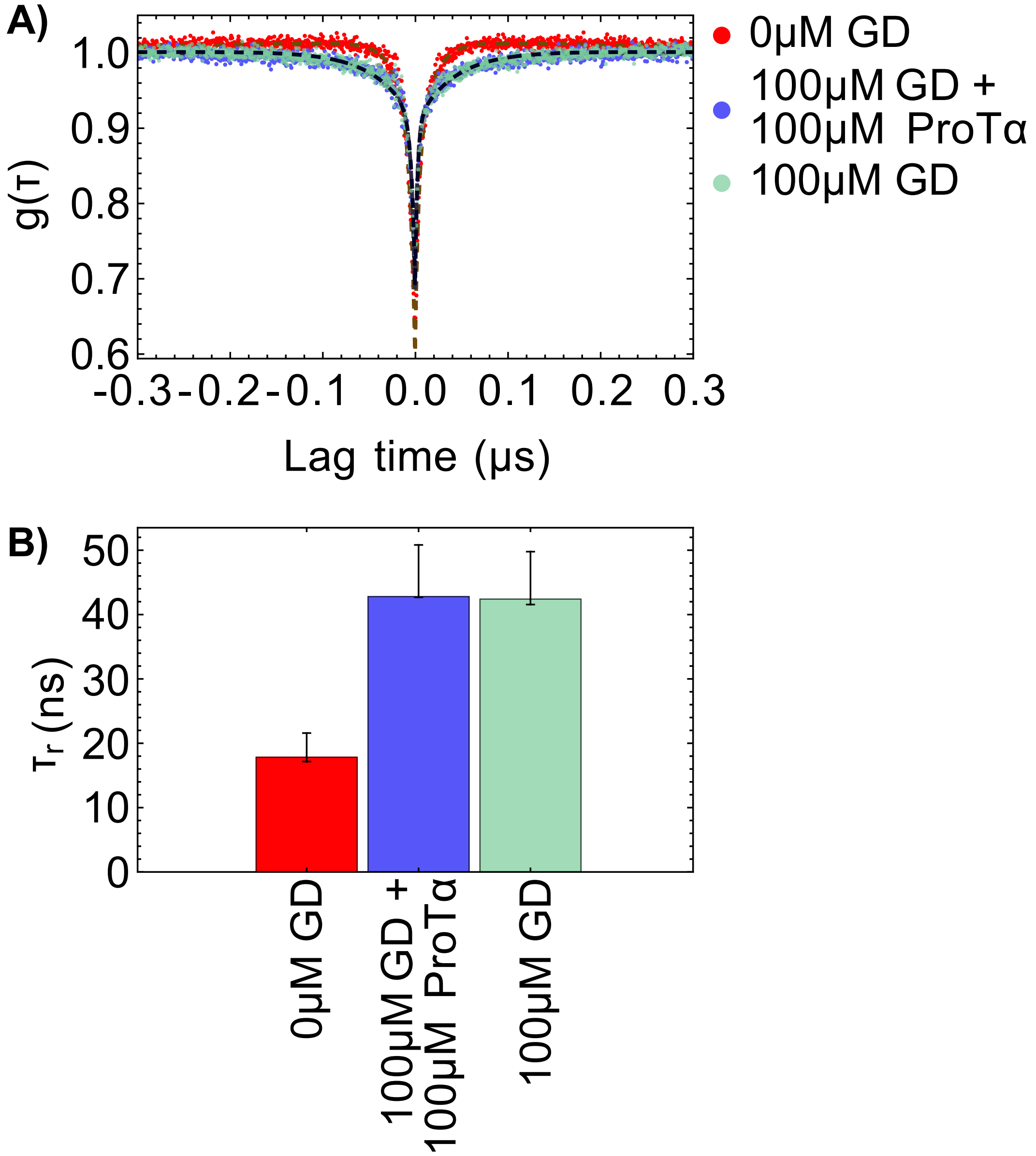

Supplementary Fig. 6*: Influence of GD binding on the chain dynamics of ProTα from nsFCS.* ***A)*** *Donor-acceptor crosscorrelations from nsFCS of fluorescently labeled ProTα in the absence of GD (yellow), in the presence of 100 μM GD (dark purple), and in the presence of 100 μM GD and 100 μM unlabeled ProTα (light purple).* ***B)*** *Comparison of resulting reconfiguration times, τ_r, obtained from the fit, with error bars reflecting the systematic uncertainty in the Förster radius of 7%* [4]*.*

Related to Figure 3.

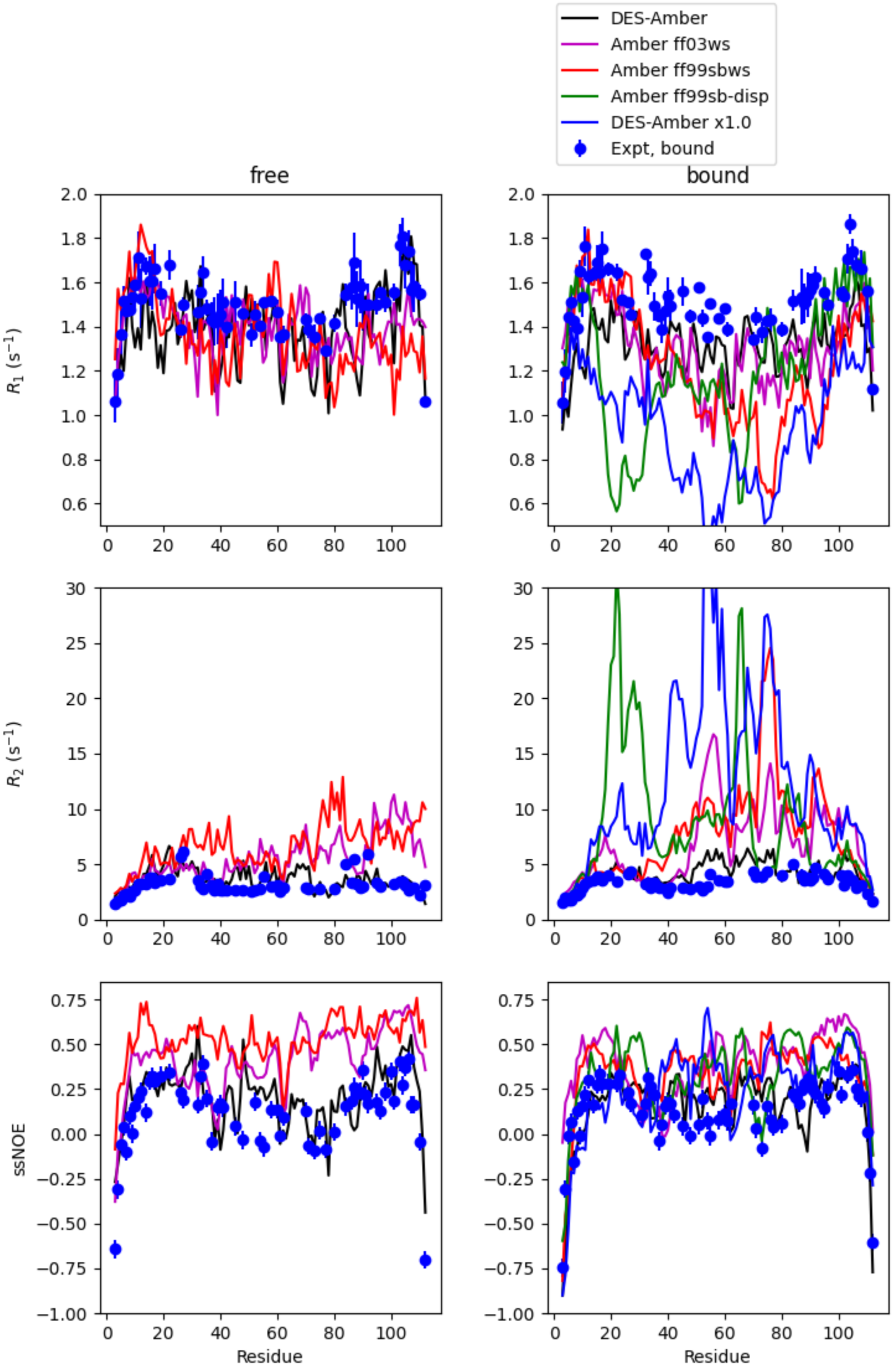

***Supplementary Fig.** 7: Comparison of experimentally determined relaxation rates for free ^{15}N-ProTα (left column) and GD bound ^{15}N-ProTα (right column, equimolar ratio) with the relaxation rates determined from molecular dynamics simulation for several different force fields (see legend). Erros bars of the experimental data are standard errors of the fits.*

Related to Figure 3.

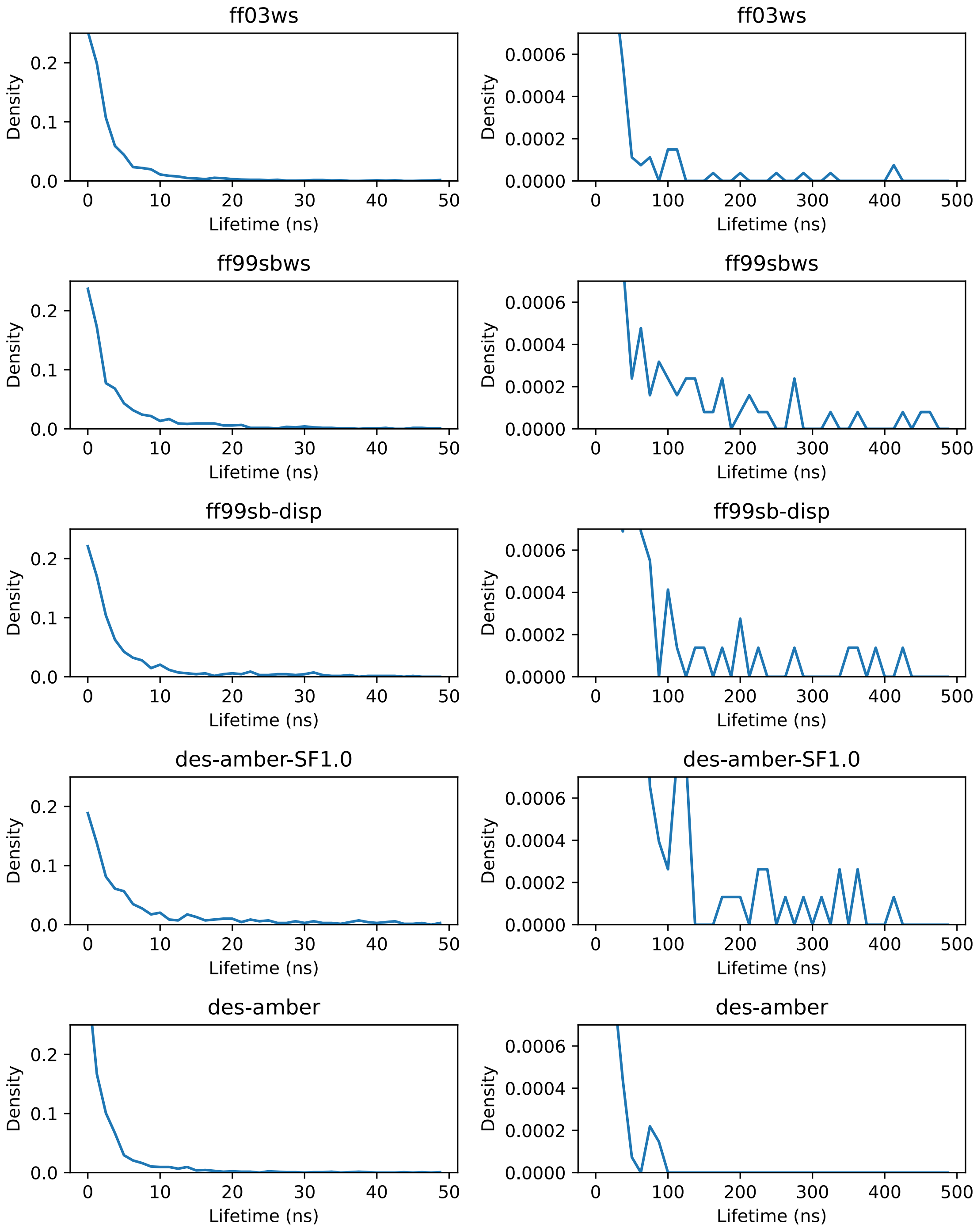

Supplementary Fig. 8*: Distributions of lifetimes of intermolecular contacts from GD-ProTα simulations using different forcefields (labeled at the top of plots). Left column shows short-lifetime distribution while right column shows the long-lifetime tail of each distribution.*

Related to Figure 3.

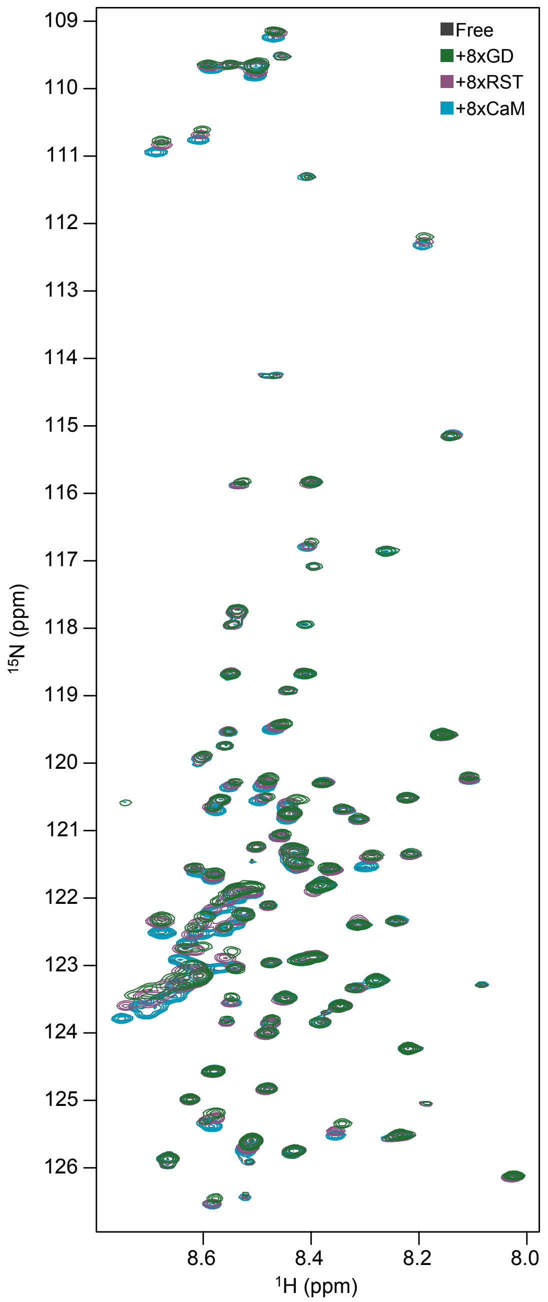

Supplementary Fig. 9: *^{15}N-HSQC spectra of ^{15}N- ProTα in its free state (black) or added 8 molar ratio of either GD (green), RST (purple) or CaM (light blue).*

Related to Figure 3.

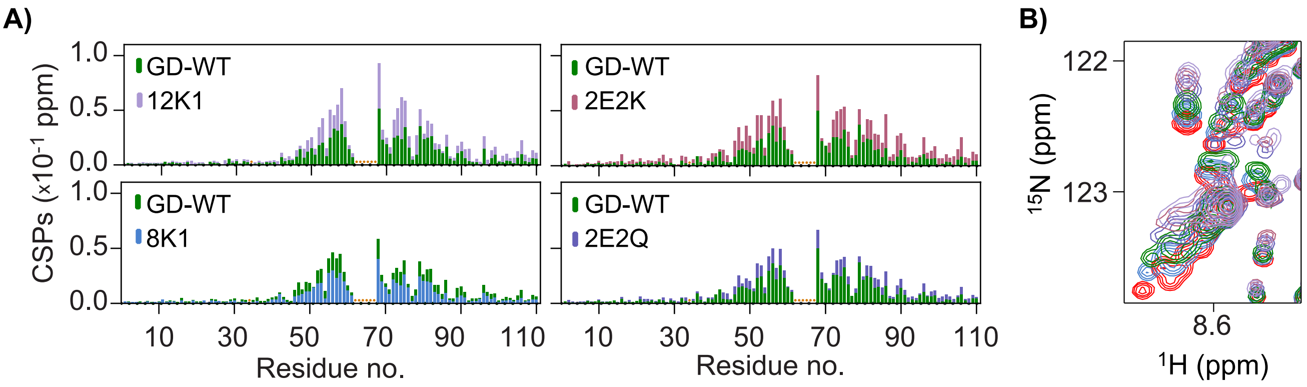

Supplementary Fig. 10*: **A**) Backbone amide chemical shift perturbations (CSPs) of ^{15}N-ProTα upon addition of GD-WT (green) or GD net charge variants (grey, see Tab. 1) at 4x molar ratio, plotted against residue number. Orange '*' highlight unassigned residues. **B**) Trajectories of the CSPs of ^{15}N-ProTα induced by the different GD variants in A), same color sheme, free ^{15}N-ProTα in red.*

Related to Figure 4.

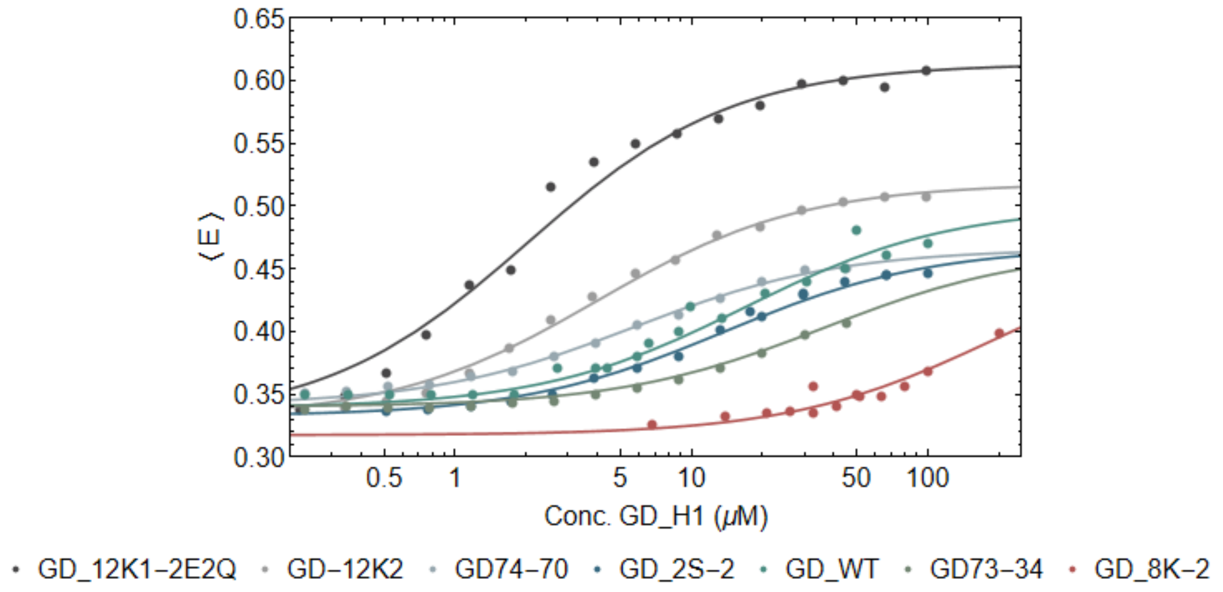

Supplementary Fig. 11*: smFRET measurements with fits to binding isotherms (see Methods) for determining K_{D}s of GD variants binding to ProTα.*

Related to Figure 4.

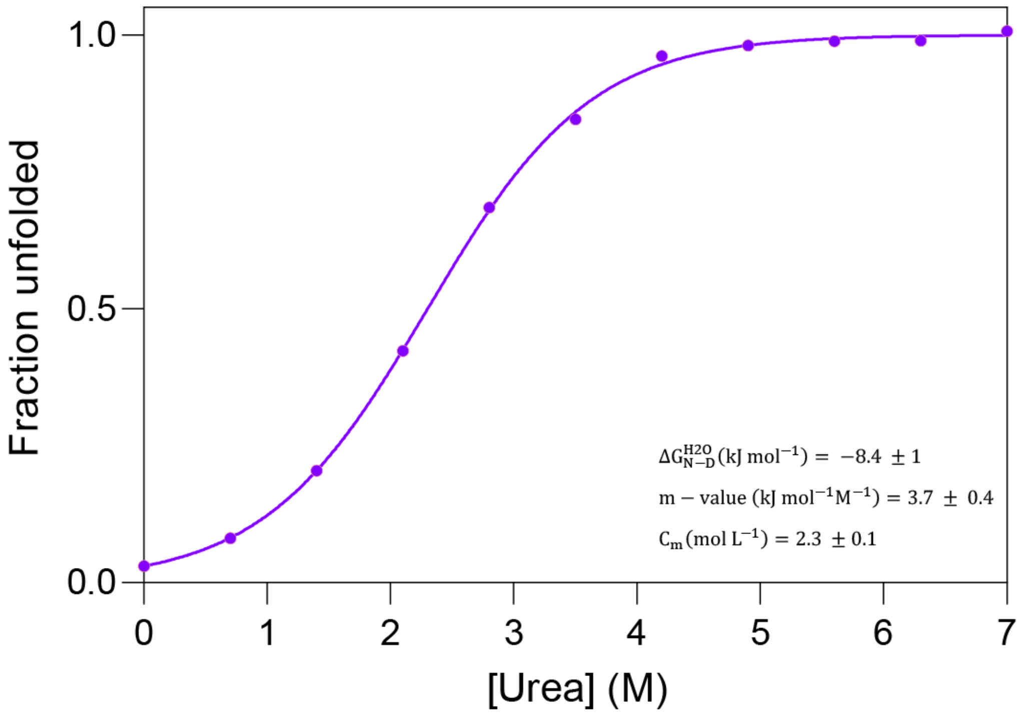

Supplementary Fig. 12*: Urea-induced unfolding of GD-WT measured by far-UV CD spectroscopy from changes in* Θ_{222nm} *as a function of urea concentration with fit (see Methods).*

Related to Figure 4.

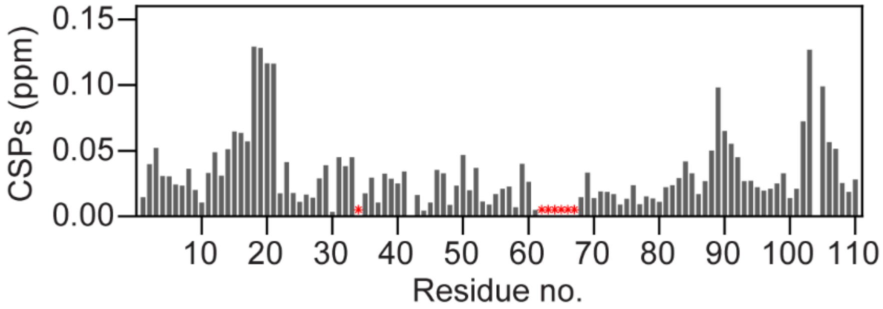

Supplementary Fig. 13*: Backbone amide chemical shift perturbations (CSPs) of free* ^{15}*N-ProTα upon addition of 4 M urea (free* ^{15}*N-ProTα (0M urea) - free* ^{15}*N-ProTα (4M urea)), plotted against residue number. Red '*' highlight unassigned residues. Large CSPs are seen in the three regions where ProTα has positive charges and are likely linked to faster exchange rate with the solvent*[5]*.*

Related to Figure 4.

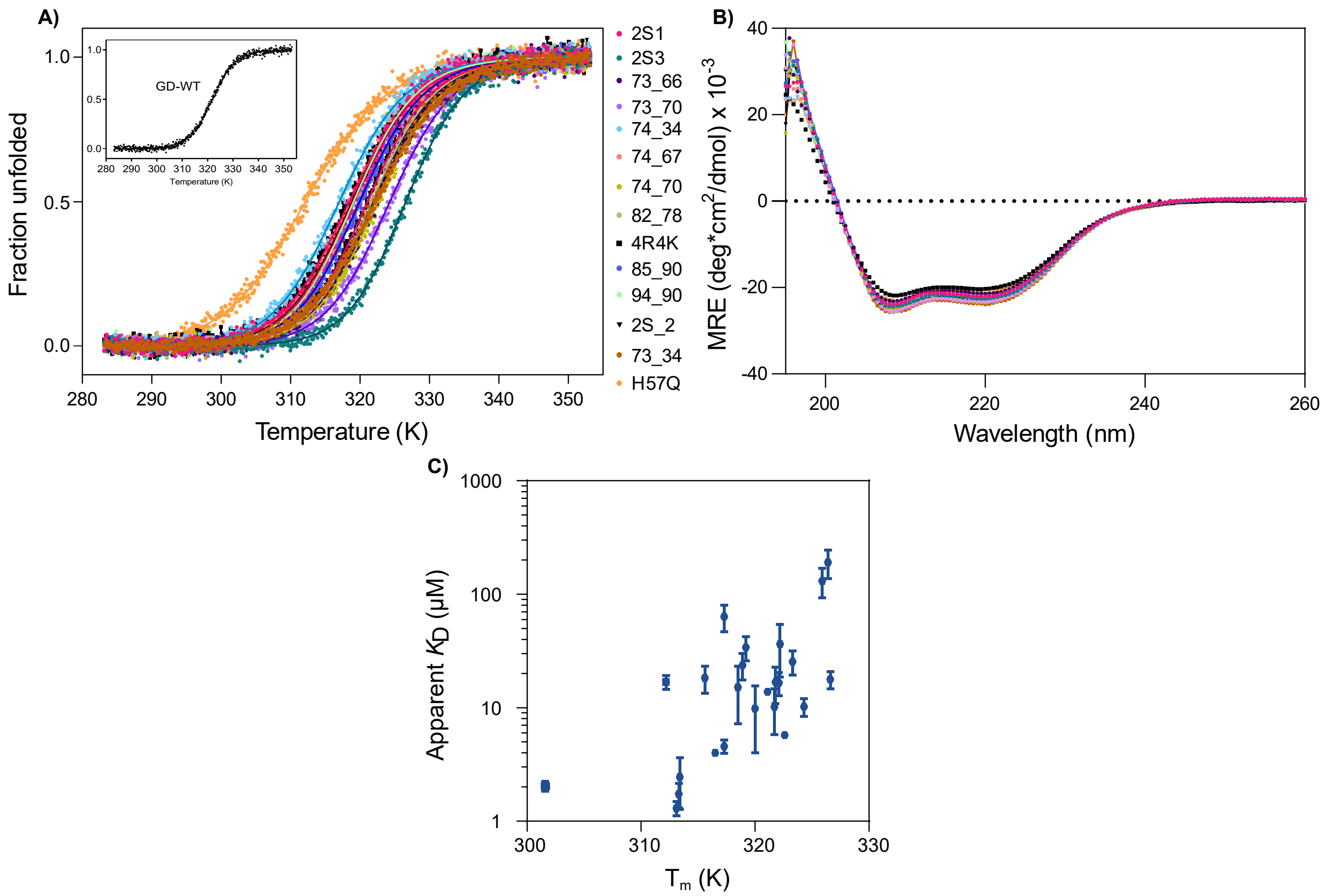

Supplementary Fig. 14: *Structure analyses of the GD charge swap variants.* ***A)*** *Thermal denaturation measured by far-UV CD spectroscopy monitoring changes in Θ_{222nm} as a function of temperature for GD-WT (insert) and GD variants (according to color code) and the corresponding fits. All data were normalized using the linear relations for the pre-and post-transition slopes representing the folded and unfolded states, respectively, obtained from the fits.* ***B)*** *Far-UV CD spectra of the same charge swap variants, same color code as in A. All data were acquired with a protein concentration of 20 µM and an ionic strength of 165 mM, pH 7.4.* ***C)*** *Correlation between melting temperature and apparent K_D measured by smFRET. Erros bars are standard errors of the fits.*

Related to Figure 5

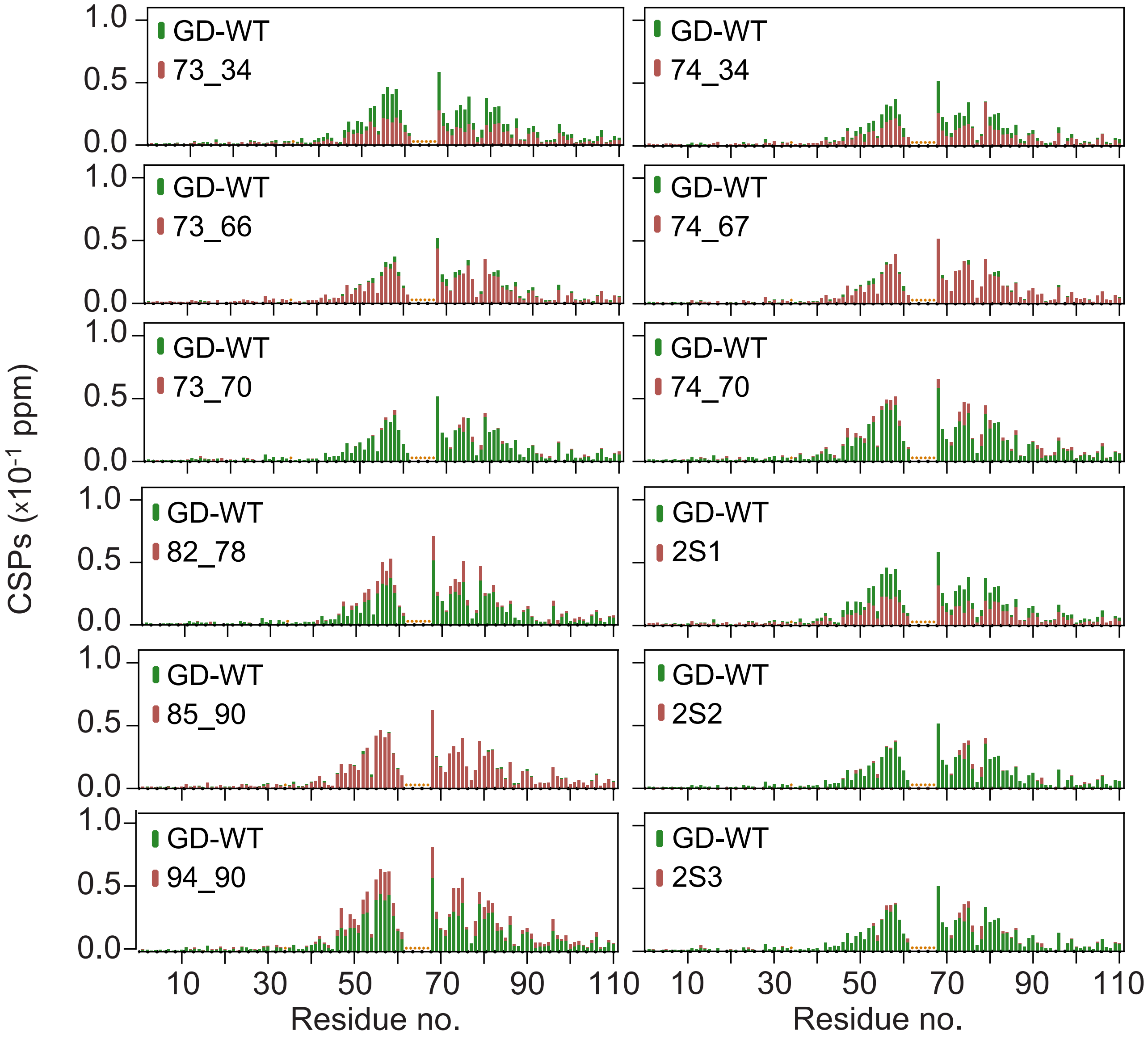

Supplementary Fig. 15: *Backbone amide chemical shift perturbations (CSPs) of* ^{15}*N-ProTα upon addition of GD-WT (green) or GD charge swap variants (red, see table 1) at a molar ratio of 1:4, plotted against residue number. Orange '*' highlight unassigned residues.*

Related to Figure 5

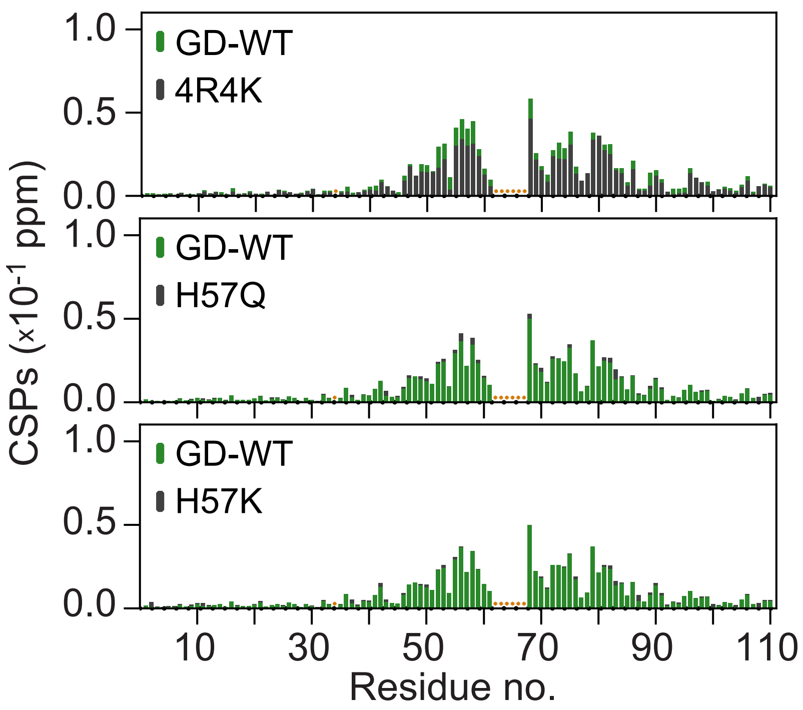

Supplementary Fig. 16: *Backbone amide chemical shift perturbations (CSPs) of ^{15}N-ProTα upon addition of GD-WT (green) or GD variant (black, see table 1) at a molar ratio of 1:4, plotted against residue number. Orange '*' highlight unassigned residues.*

Related to Figure 5.

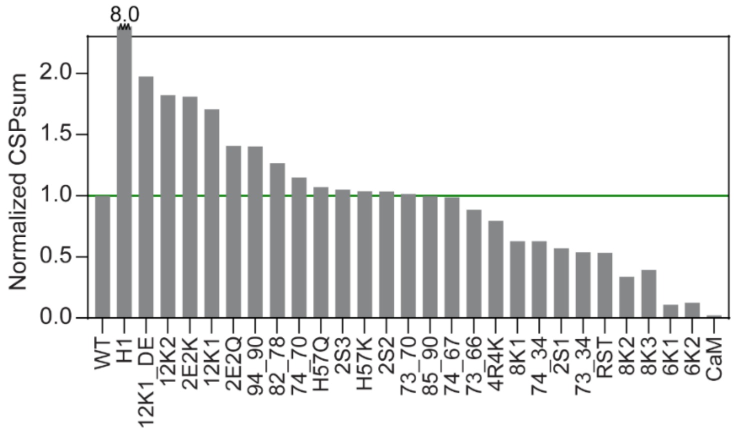

Supplementary Fig. 17: *Normalized backbone amide chemical shift perturbations (CSPsum; see methods) of ^{15}N-ProTα upon addition equimolar amounts of different RST, CaM, GD variants, GD-WT and full-length H1. The bar representing full-length H1 has been shortened for illustrative purposes.*

Related to Figure 5.

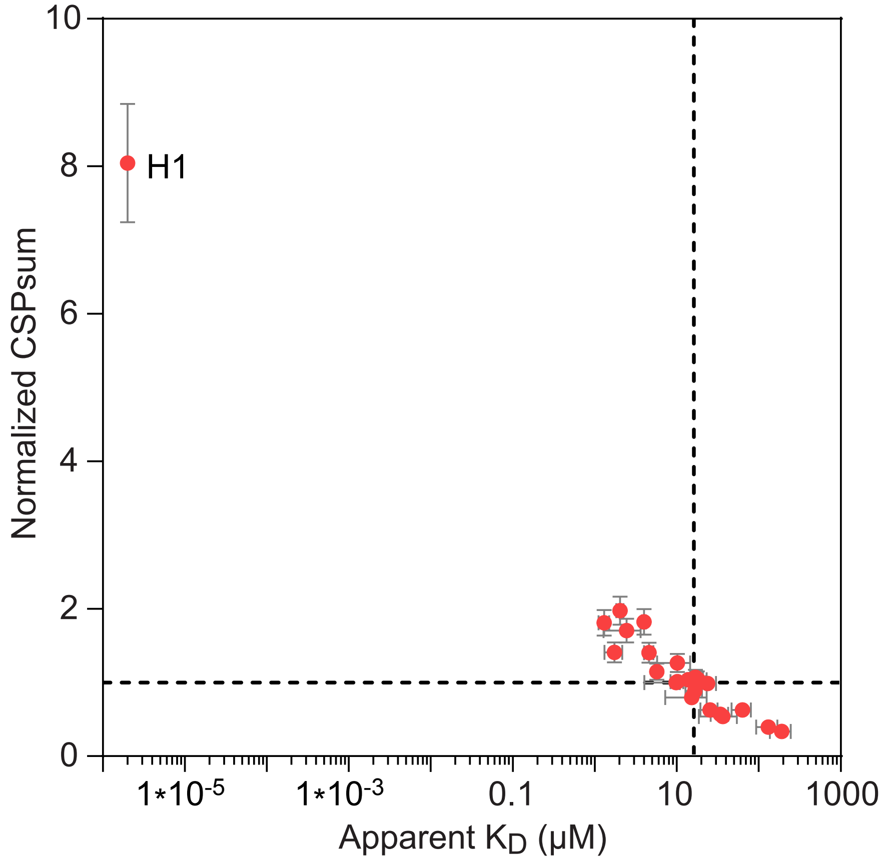

Supplementary Fig. 18*: Normalized sum of backbone amide chemical shift perturbations (CSP$_{sum}$; see methods) plotted against apparent K$_D$ for GD-WT, 23 GD variants and full-length H1. The black dotted line represents GD-WT values. Erros bars for the K$_D$s are standard errors of the fits. Erros bars for the normalized CSPsum are propagated errors from three repetition of GD WT.*

Related to Figure 5.

A)

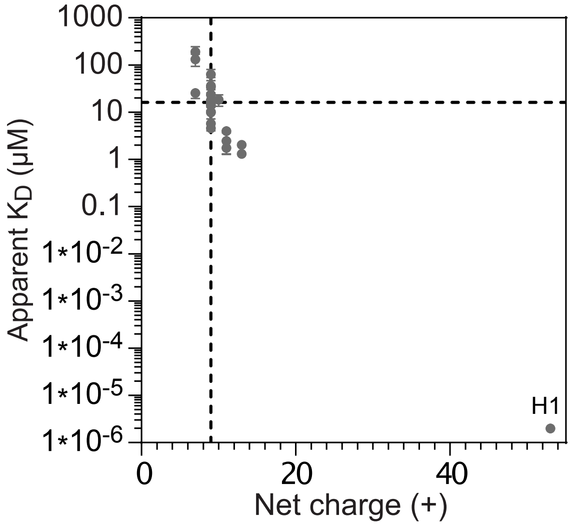

B)

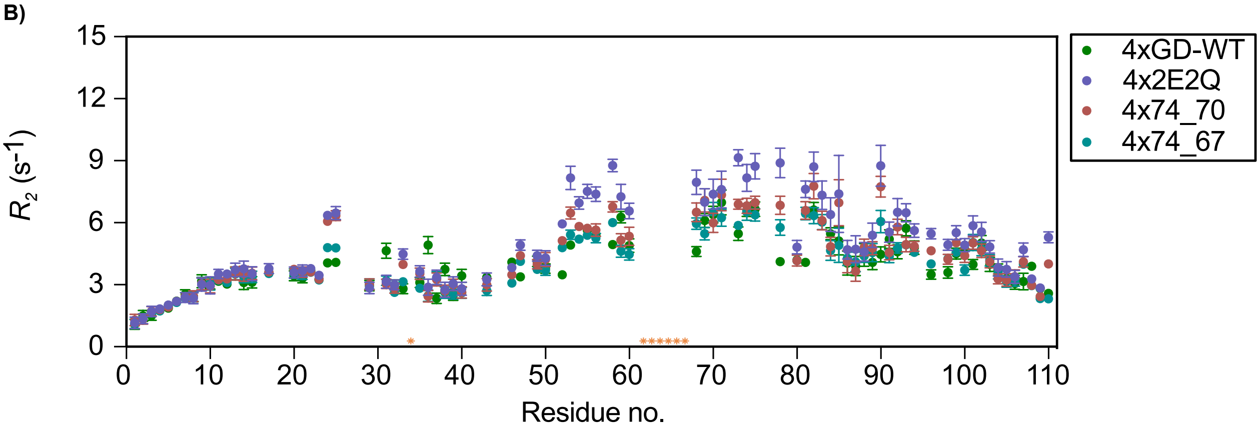

Supplementary Fig. 19*: A) Apparent K_Ds for ProTα plotted against net charge of GD-WT, 23 GD variants and full-length H1. The black dotted line indicates GD-WT values.* ***B****) R_2 values of ProTα mixed at 1:4 molar ratio with GD-WT, GD 74_70, GD 74_67 and GD 2E2Q. Error bars are standard errors from the fits. Missing data points are due to signal overlap or proline residues.*

Related to Figure 5.

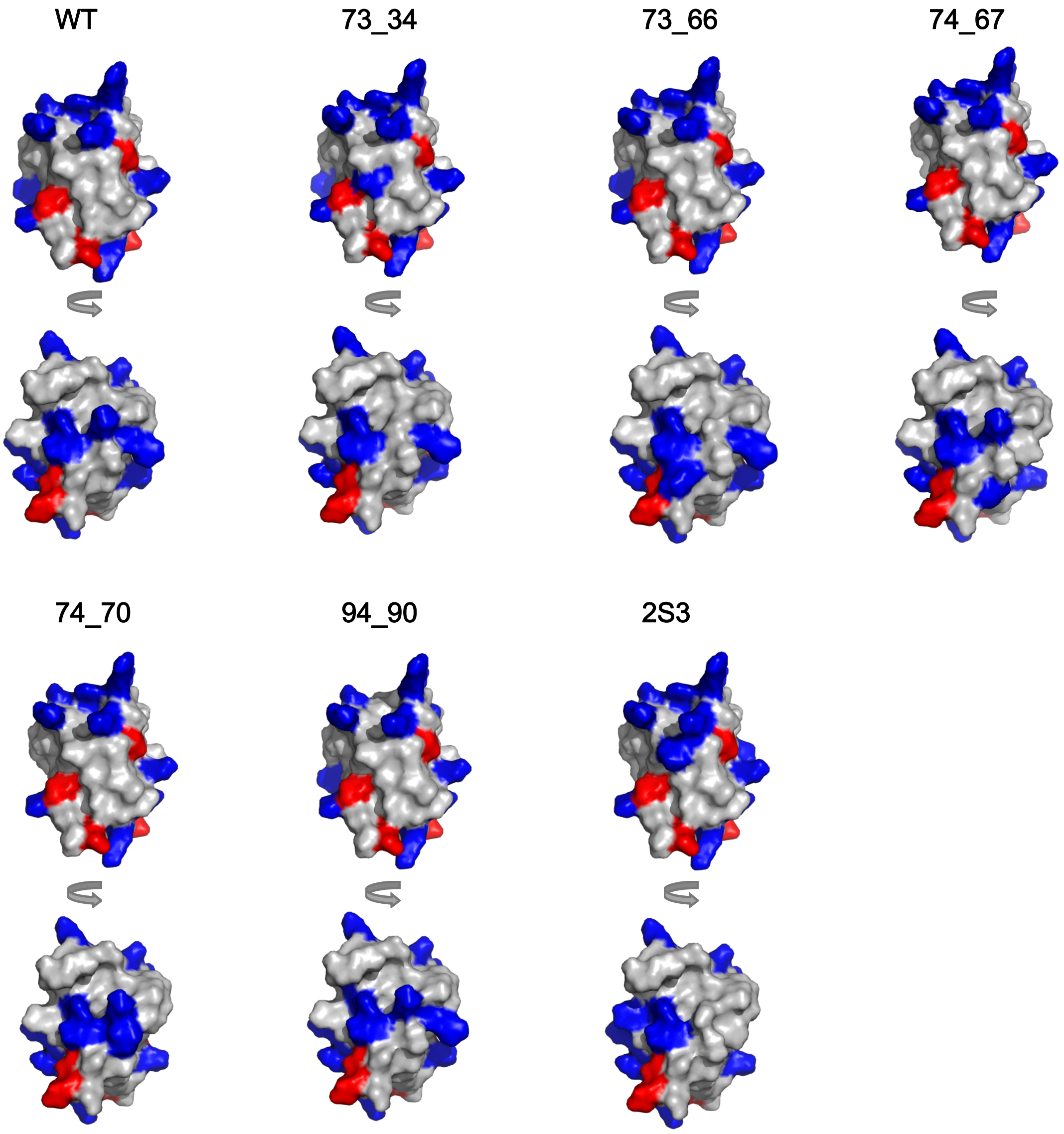

Supplementary Fig. 20: *Illustration of charge clustering in GD charge clustering variants. Shown here are the seven different +9 variants of GD including GD-WT in two orientations each.*

Related to Figure 5.

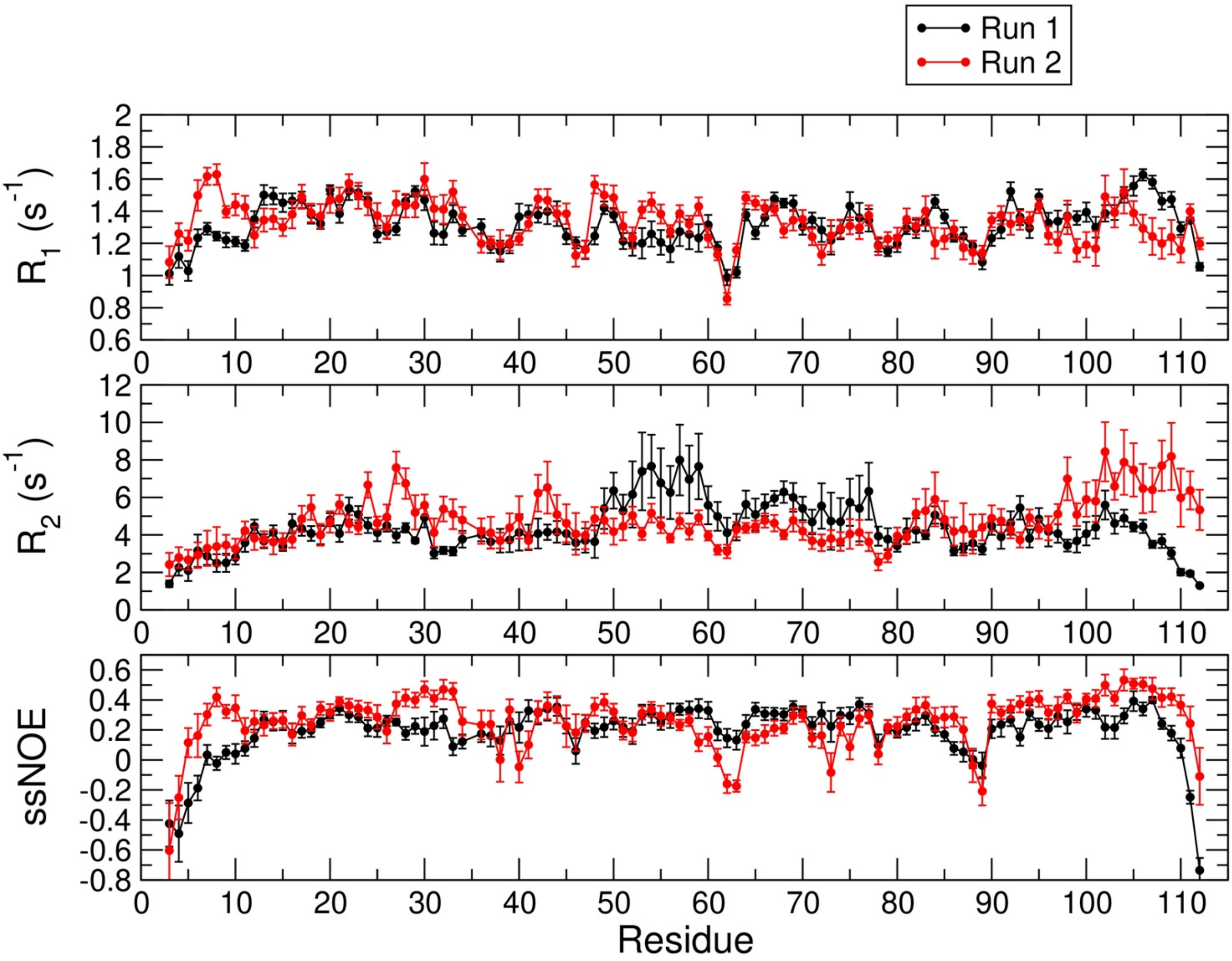

***Supplementary Fig. 21**: Comparison of relaxation parameters calculated from two independent simulations of the GD:ProTα complex with the DES-Amber force field.*

Related to Figure 5.

SUPPLEMENTARY TABLES

Supplementary Table 1: *K_D values at higher stoichiometries from the coarse-grained simulations*

		Excess titration	Stoichiometric titration
1:1	$\langle E \rangle_{ProTa:GD}$	0.50 ± 0.01	0.475 ± 0.004
	$K_D\ (\mu M)$	18 ± 6	15 ± 2
1:4	$\langle E \rangle_{ProTa:GD1}$	0.51 ± 0.04	0.4754 ± 0.0003
	$\langle E \rangle_{ProTa:GD2}$	0.53 ± 0.04	0.4950 ± 0.0003
	$\langle E \rangle_{ProTa:GD3}$	0.54 ± 0.05	0.5077 ± 0.0003
	$\langle E \rangle_{ProTa:GD4}$	0.54 ± 0.05	0.5085 ± 0.0003
	$K_{D_{ProTa:GD1}}\ (\mu M)$	18 ± 7	15.2 ± 0.6
	$K_{D_{ProTa:GD2}}\ (\mu M)$	$(0 \pm 4)10^9$	$(0 \pm 4)10^9$
	$K_{D_{ProTa:GD3}}\ (\mu M)$	$(0.0 \pm 3)10^8$	$(0.0 \pm 3)10^8$
	$K_{D_{ProTa:GD4}}\ (\mu M)$	$(2 \pm 2)10^7$	$(1 \pm 2)10^8$

Supplementary Table 2: *Summary of simulations performed (detailed setups provided at https://dx.doi.org/10.5281/zenodo.11106958)*

System	Force Field	# Atoms	# replicates	Trajectory lengths (μs)
ProTα + GD	Amber ff99SBws[6]	492,885	1	1.60
ProTα + GD	Amber ff03ws[6]	492,885	1	4.06
ProTα + GD	Amber-99SB-disp[7]	492,885	1	1.02
ProTα + GD	DES-Amber-SF1.0[8]	495,661	1	0.83
ProTα + GD	DES-Amber[8]	495,629	2	1.33, 0.86
ProTα	Amber ff99SBws[6]	496,184	1	0.58
ProTα	Amber ff03ws[6]	493,448	1	0.89
ProTα	DES-Amber[8]	496,188	1	0.43
GD	Amber ff99SBws[6]	21,419	10	1.0 each
GD	Amber ff03ws	21,423	10	1.0 each
GD	DES-Amber[8]	21,479	10	1.0 each
ProTα	Coarse-grained[9]	112	10	511
ProTα + GD	Coarse-grained[9]	187	10	30
ProTα + 2 GDs	Coarse-grained[9]	262	10	30
ProTα + 3 GDs	Coarse-grained[9]	337	10	30
ProTα + 4 GDs	Coarse-grained[9]	412	10	30
ProTα + 5 GDs	Coarse-grained[9]	487	10	30
ProTα + 6 GDs	Coarse-grained[9]	562	10	30
ProTα + 7 GDS	Coarse-grained[9]	637	10	30
ProTα + 20 GDS	Coarse-grained[9]	1612	6	20.7
Prota + WT GD 260 K	Coarse-grained[9]	187	28 umbrellas	5.4
Prota + WT GD 280 K	Coarse-grained[9]	187	28 umbrellas	4.9
Prota + WT GD 300 K	Coarse-grained[9]	187	28 umbrellas	5.1
Prota + WT GD 320 K	Coarse-grained[9]	187	28 umbrellas	5.3
Prota+GD73-34 260 K	Coarse-grained[9]	187	28 umbrellas	5.0
Prota+GD73-34 280 K	Coarse-grained[9]	187	28 umbrellas	4.9

Prota+GD73-34 300 K	Coarse-grained[9]	187	28 umbrellas	5.1
Prota+GD73-34 320 K	Coarse-grained[9]	187	28 umbrellas	5.2
Prota+GD73-66 260 K	Coarse-grained[9]	187	28 umbrellas	4.8
Prota+GD73-66 280 K	Coarse-grained[9]	187	28 umbrellas	4.9
Prota+GD73-66 300 K	Coarse-grained[9]	187	28 umbrellas	5.1
Prota+GD73-66 320 K	Coarse-grained[9]	187	28 umbrellas	5.3
Prota+GD74-34 260 K	Coarse-grained[9]	187	28 umbrellas	4.7
Prota+GD74-34 280 K	Coarse-grained[9]	187	28 umbrellas	4.9
Prota+GD74-34 300 K	Coarse-grained[9]	187	28 umbrellas	5.1
Prota+GD74-34 320 K	Coarse-grained[9]	187	28 umbrellas	5.2
Prota+GD74-67 260 K	Coarse-grained[9]	187	28 umbrellas	4.8
Prota+GD74-67 280 K	Coarse-grained[9]	187	28 umbrellas	4.9
Prota+GD74-67 300 K	Coarse-grained[9]	187	28 umbrellas	5.1
Prota+GD74-67 320 K	Coarse-grained[9]	187	28 umbrellas	5.3
Prota+GD74-70 260 K	Coarse-grained[9]	187	28 umbrellas	5.5
Prota+GD74-70 280 K	Coarse-grained[9]	187	28 umbrellas	4.9
Prota+GD74-70 300 K	Coarse-grained[9]	187	28 umbrellas	5.1
Prota+GD74-70 320 K	Coarse-grained[9]	187	28 umbrellas	5.2
Prota+GD94-90 260 K	Coarse-grained[9]	187	28 umbrellas	5.4
Prota+GD94-90 280 K	Coarse-grained[9]	187	28 umbrellas	4.9
Prota+GD94-90 300 K	Coarse-grained[9]	187	28 umbrellas	5.1
Prota+GD94-90 320 K	Coarse-grained[9]	187	28 umbrellas	5.3
Prota+GD CC 260 K	Coarse-grained[9]	187	28 umbrellas	5.0
Prota+GD CC 280 K	Coarse-grained[9]	187	28 umbrellas	4.9
Prota+GD CC 300 K	Coarse-grained[9]	187	28 umbrellas	5.1
Prota+GD CC 320 K	Coarse-grained[9]	187	28 umbrellas	5.2

Supplementary References

1. Record, M. T., Anderson, C. F. & Lohman, T. M. Thermodynamic analysis of ion effects on the binding and conformational equilibria of proteins and nucleic acids: the roles of ion association or release, screening, and ion effects on water activity. *Q Rev Biophys* **11**, 103–178 (1978).

2. Martinsen, J. H. *et al.* Structure, dynamics, and stability of the globular domain of human linker histone H1.0 and the role of positive charges. *Protein Sci* **31**, 918–932 (2022).

3. Kneller, J. M., Lu, M. & Bracken, C. An effective method for the discrimination of motional anisotropy and chemical exchange. *J Am Chem Soc* **124**, 1852–1853 (2002).

4. Hellenkamp, B. *et al.* Precision and accuracy of single-molecule FRET measurements-a multi-laboratory benchmark study. *Nat Methods* **15**, 669–676 (2018).

5. Dass, R., Corlianò, E. & Mulder, F. A. A. The contribution of electrostatics to hydrogen exchange in the unfolded protein state. *Biophys J* **120**, 4107–4114 (2021).

6. Best, R. B., Zheng, W. & Mittal, J. Balanced Protein–Water Interactions Improve Properties of Disordered Proteins and Non-Specific Protein Association. *J Chem Theory Comput* **10**, 5113–5124 (2014).

7. Robustelli, P., Piana, S. & Shaw, D. E. Developing a molecular dynamics force field for both folded and disordered protein states. *Proceedings of the National Academy of Sciences* **115**, (2018).

8. Piana, S., Robustelli, P., Tan, D., Chen, S. & Shaw, D. E. Development of a Force Field for the Simulation of Single-Chain Proteins and Protein–Protein Complexes. *J Chem Theory Comput* **16**, 2494–2507 (2020).

9. Borgia, A. *et al.* Extreme disorder in an ultrahigh-affinity protein complex. *Nature* **555**, 61–66 (2018).
