# Supplementary material for: Role of charges in a dynamic disordered complex between an IDP and a folded domain
Source: Nat Commun. 2025 Apr 4;16:3242. doi: 10.1038/s41467-025-58374-5 (PMC11971343; doi:10.1038/s41467-025-58374-5)
Supplement: Supplementary file 2 — Description of Additional Supplementary Files [file 41467_2025_58374_MOESM2_ESM.pdf]

Description of Additional Supplementary Files

File name: Supplementary Movie 1
Description: Movie showing transient interactions between the GD (blue) and prothymosin alpha (red) during simulations with DES-Amber force field. Proteins are shown in liquorice representation.
