# Supplementary material for: Role of charges in a dynamic disordered complex between an IDP and a folded domain
Source: Nat Commun. 2025 Apr 4;16:3242. doi: 10.1038/s41467-025-58374-5 (PMC11971343; doi:10.1038/s41467-025-58374-5)
Supplement: Supplementary file 4 — Reporting Summary [file 41467_2025_58374_MOESM4_ESM.pdf]

nature portfolio

Corresponding author(s): Birthe B. Kragelund

Last updated by author(s): Jun 5, 2024

Reporting Summary

Nature Portfolio wishes to improve the reproducibility of the work that we publish. This form provides structure for consistency and transparency in reporting. For further information on Nature Portfolio policies, see our Editorial Policies and the Editorial Policy Checklist.

Statistics

For all statistical analyses, confirm that the following items are present in the figure legend, table legend, main text, or Methods section.

n/a	Confirmed	
☐	☒	The exact sample size (*n*) for each experimental group/condition, given as a discrete number and unit of measurement
☐	☒	A statement on whether measurements were taken from distinct samples or whether the same sample was measured repeatedly
☒	☐	The statistical test(s) used AND whether they are one- or two-sided<br>*Only common tests should be described solely by name; describe more complex techniques in the Methods section.*
☒	☐	A description of all covariates tested
☒	☐	A description of any assumptions or corrections, such as tests of normality and adjustment for multiple comparisons
☐	☒	A full description of the statistical parameters including central tendency (e.g. means) or other basic estimates (e.g. regression coefficient) AND variation (e.g. standard deviation) or associated estimates of uncertainty (e.g. confidence intervals)
☒	☐	For null hypothesis testing, the test statistic (e.g. *F*, *t*, *r*) with confidence intervals, effect sizes, degrees of freedom and *P* value noted<br>*Give P values as exact values whenever suitable.*
☒	☐	For Bayesian analysis, information on the choice of priors and Markov chain Monte Carlo settings
☒	☐	For hierarchical and complex designs, identification of the appropriate level for tests and full reporting of outcomes
☒	☐	Estimates of effect sizes (e.g. Cohen's *d*, Pearson's *r*), indicating how they were calculated

Our web collection on statistics for biologists contains articles on many of the points above.

Software and code

Policy information about availability of computer code

Data collection	SymphoTime 64 version 2.4 (PicoQuant) was used for single molecule data collection. NMR data were recorded at cOpenNMR (supported by the Novo Nordisk Foundation) on Bruker instruments using Topspin versions 3.6.5, 4.0.7 or 4.2.0 (Bruker Biospin). CD data were recorded on Jasco J815 using SpectraManager from Jasco Inc. MD simulations were run using GROMACS 4.6.5 and GROMACS 2018.3, available under open source license from https://www.gromacs.org.
Data analysis	Fretica, a custom add-on package for Mathematica v.12.3 (Wolfram Research) was used for the analysis of single-molecule fluorescence data and is available at https://github.com/SchulerLab. For NMR data analyses, pMDD 60, NMRPipe, CcpNMR were used, otherwise GraphPad Prism 8.4.1 and SpectraManager (Jasoc Inc). Simulations were analyzed via C code and python scripts available from https://github.com/bestlab.

For manuscripts utilizing custom algorithms or software that are central to the research but not yet described in published literature, software must be made available to editors and reviewers. We strongly encourage code deposition in a community repository (e.g. GitHub). See the Nature Portfolio guidelines for submitting code & software for further information.

Data

Policy information about availability of data

All manuscripts must include a data availability statement. This statement should provide the following information, where applicable:

- Accession codes, unique identifiers, or web links for publicly available datasets
- A description of any restrictions on data availability
- For clinical datasets or third party data, please ensure that the statement adheres to our policy

Molecular simulation input files and trajectories generated for this study are provided on Zenodo at https://dx.doi.org/10.5281/zenodo.11106958.
Chemical shifts are available at BioMagResBank accession codes 27215 (ProTα) and 34318 (GD), respectively.
All NMR data are available on request.

Research involving human participants, their data, or biological material

Policy information about studies with human participants or human data. See also policy information about sex, gender (identity/presentation), and sexual orientation and race, ethnicity and racism.

Reporting on sex and gender	Not relevant
Reporting on race, ethnicity, or other socially relevant groupings	Not relevant
Population characteristics	Not relevant
Recruitment	Not relevant
Ethics oversight	Not relevant

Note that full information on the approval of the study protocol must also be provided in the manuscript.

Field-specific reporting

Please select the one below that is the best fit for your research. If you are not sure, read the appropriate sections before making your selection.

☒ Life sciences ☐ Behavioural & social sciences ☐ Ecological, evolutionary & environmental sciences

For a reference copy of the document with all sections, see nature.com/documents/nr-reporting-summary-flat.pdf

Life sciences study design

All studies must disclose on these points even when the disclosure is negative.

Sample size	The sample size for all free diffusion FRET experiments was always > 4000 freely diffusing molecules. For the nanosecond fluorescence correlation time experiments, fluorescent photons from more than 300,000 fluorescently labeled freely diffusing molecule observed for at least 30 hours. The sample sizes were found to satisfactorily describe the conformational distributions and dynamics in the ensembles. For NMR, the sample sizes were based on previous comparable experiments. Each all-atom molecular simulation trajectory was run for at least 1 microsecond; details are provided in the supporting information. Coarse-grained umbrella sampling simulations were run for 2 microseconds
Data exclusions	Bursts arising from freely diffusing molecules with only a single fluorophore and with < 50 photons were excluded from analysis. More details on Data exclusion and selection can be found in the Methods section. For NMR analyses and protein stability measurements, data were not excluded. Data were not excluded for MD simulation analysis, except for the umbrella sampling simulations where the first 1 microsecond was discarded as equilibration.
Replication	The overall reproducibility of the main experiments was confirmed by making multiple independent measurements. For NMR analyses, data were recorded once except for the relaxation data, where data were recorded in triplicate on the same sample to extract error bars. For the force field used in the main text, two independent simulations were performed.
Randomization	No randomization was used
Blinding	Experiments were not blinded.

Reporting for specific materials, systems and methods

We require information from authors about some types of materials, experimental systems and methods used in many studies. Here, indicate whether each material, system or method listed is relevant to your study. If you are not sure if a list item applies to your research, read the appropriate section before selecting a response.

Materials & experimental systems

n/a	Involved in the study	
x		Antibodies
x		Eukaryotic cell lines
x		Palaeontology and archaeology
x		Animals and other organisms
x		Clinical data
x		Dual use research of concern
x		Plants

Methods

n/a	Involved in the study	
x		ChIP-seq
x		Flow cytometry
x		MRI-based neuroimaging

Plants

Seed stocks	Not relevant
Novel plant genotypes	Not relevant
Authentication	Not relevant
